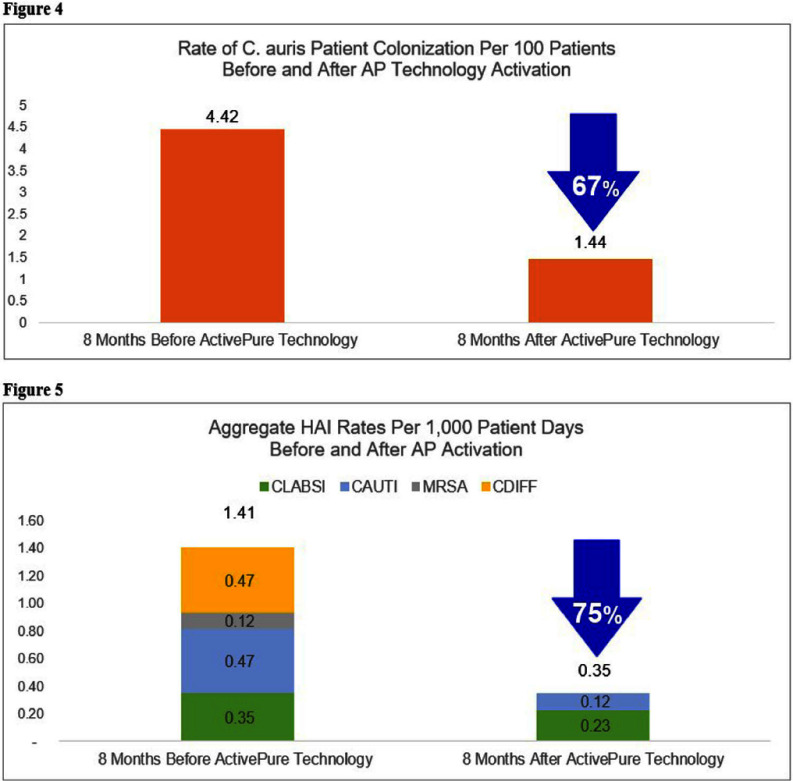# Advanced Photohydrolysis Technology Reduction of C. auris, fungi, & aerobic bacteria CFUs with Impact on C. auris Colonizations & HAIs

**DOI:** 10.1017/ash.2025.325

**Published:** 2025-09-24

**Authors:** Kimberly Trosch, Amy Carenza, Uzoamaka Obiekwe, Kirk Huslage, Deborah Birx

**Affiliations:** 1 ActivePure Technologies; 2 ActivePure; 3 LifeBridge Health; 4 Lifebridge Health

## Abstract

**Background:** The continued rise of antimicrobial resistance (AMR) and reduction of pharmacological options has created an urgent need for new countermeasures to prevent the spread of multidrug-resistant organisms (MDROs) (1). Infections caused by the MDRO Candida auris (C. auris) have been proliferating since its discovery in 2009 and are associated with high mortality and AMR (2). Attention must be given to pathogen reservoirs, including surfaces of floors, which have an underappreciated potential to transfer pathogens to hands from objects in contact with the floor (3). **Methods:** An experimental study to explore the impact of advanced photohydrolysis (AP) technology on colony forming units (CFUs) of aerobic bacteria, fungi, and C. auris inside a hospital unit with active C. auris infections was performed from September 2023 to January 2024. Baseline pre-activation samples were compared to post-activation samples, which occurred every four weeks on Tuesday mornings prior to daily environmental services (EVS) cleaning. Patient outcomes of C. auris colonization and HAI rates were compared 8 months before and after AP technology installation. C. auris point-prevalence testing was performed during the 16-month observation period according to health department protocols and HAIs were evaluated by the same infection preventionist and defined by NHSN standards for catheter associated urinary tract infections (CAUTIs), central line associated bloodstream infections (CLABSIs), Clostridioides difficile (CDIFF), and methicillin resistant Staphylococcus aureus (MRSA) (4). **Results:** Pairwise comparisons with a Bonferroni correction found median floor fungal CFUs achieved a statistically significant reduction of 99% (p=0.11) from Baseline to Post-Activation #4 (Figure 1). Aerobic bacteria decreased 98% (Figure 2); C. auris by 66% (Figure 3) but did not achieve statistical significance. The rate of patient C. auris colonization after admission decreased 67% (Figure 4) and aggregate HAI rates decreased 75% (Figure 5). **Additional Findings:** High-touch surface samples showed reductions in mean CFUs of aerobic bacteria by 82% (6,456 to 1,161) and fungi by 99% (2,089 to 15). C. auris was reduced by 100% from Baseline to Post-Activation #3 (41 to 0) but increased in Post-Activation #4 due to two positive samples in a patient room with an active C. auris infection. Both samples tested under 500 CFU/cm2, which is associated with lower infection risk from environmental bioburden (5). **Conclusion:** These findings of significant reductions in microbial bioburden and abated pathogen acquisition demonstrate the positive impact that AP continuous disinfection technology can have on the environment and patient outcomes, without additional skilled labor or increases in cleaning and disinfection practices.

**Figure:**
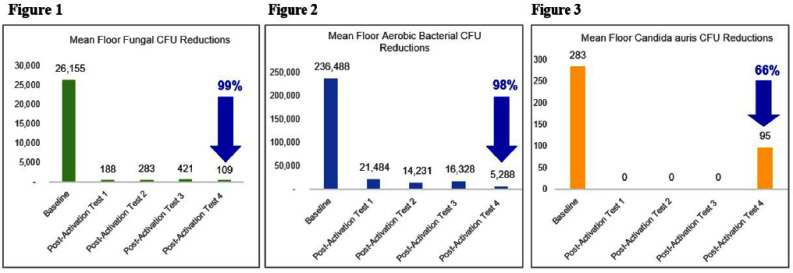

**Figure:**